# Oligomeric Amyloid-β Peptide on Sialylic Lewisx–Selectin Bonding at Cerebral Endothelial Surface

**DOI:** 10.5195/cajgh.2014.150

**Published:** 2014-12-12

**Authors:** Sholpan Askarova, Grace Y. Sun, Gerald A. Meininger, James Lee

**Affiliations:** 1 Center for Life Sciences, Nazarbayev University, Astana, Kazakhstan; 2 Department of Biochemistry, University of Missouri, USA; 3 Dalton Cardiovascular Research Center, University of Missouri, USA; 4 Department of Biological Engineeering, University of Missouri, USA

**Keywords:** Alzheimer’s disease, statins, brain inflamation, treatment

## Abstract

**Introduction:**

Alzheimer’s disease (AD) is a chronic neurodegenerative disorder, which affects approximately 10% of the population aged 65 and 40% of people over the age 80. Currently, AD is on the list of diseases with no effective treatment. Thus, the study of molecular and cellular mechanisms of AD progression is of high scientific and practical importance. In fact, dysfunction of the blood-brain barrier (BBB) plays an important role in the onset and progression of the disease. Increased deposition of amyloid b peptide (Aβ) in cerebral vasculature and enhanced transmigration of monocytes across the BBB are frequently observed in AD brains and are some of the pathological hallmarks of the diseases. Since the transmigration of monocytes across the BBB is both a mechanical and a biochemical process, the expression of adhesion molecules and mechanical properties of endothelial cells are the critical factors that require investigation.

**Methods:**

Because of recent advances in the biological applications of atomic force microscopy (AFM), we applied AFM with cantilever tips bio-functionalized by sLe^x^ in combination with the advanced immunofluorescent microscopy (QIM) to study the direct effects of Aβ_42_ oligomers on the selectins expression, actin polymerization, and cellular mechanical and adhesion properties in cerebral endothelial cells (mouse bEnd3 line and primary human CECs) and find a possible way to attenuate these effects.

**Results:**

QIM results showed that Aβ_42_ increased the expressions of P-selectin on the cell surface and enhanced actin polymerization. Consistent with our QIM results, AFM data showed that Aβ_42_ increased the probability of cell adhesion with sLe^x^-coated cantilever and cell stiffness. These effects were counteracted by lovstatin, a cholesterol-lowering drug. Surprisingly, the apparent rupture force of sLe^x^-selectin bonding was significantly lower after treatment with Aβ_42_, as compared with the control (i.e. no treatment). Similar results were also obtained when cells were treated with latruculin A (F-actin-disrupting drug). These results suggest that the decrease in the apparent rupture force of sLe^x^-selectin bonding is the consequence of the dissociation of adhesion between the cytoskeleton and the bilayer membrane induced by Aβ_42_. The major causes of excess mortality in the first group were neoplams (30.6%), hypertension (23.8%), and myocardial infarction (22.6%). The effects of radiation influenced mortality in the second group were 2–2.5 times lower than the first group.

**Conclusion:**

The studies of the effects of Aβ_42_ on the adhesion properties of cerebral endothelial cells and how pharmacological agents (e.g. statin) counteract these effects should prove to provide insights into the mechanism of inflammation in Alzheimer’s brains and the design of therapeutic treatments of the disease.

